# Prediction of Pharmacokinetics of IDP-73152 in Humans Using Physiologically-Based Pharmacokinetics

**DOI:** 10.3390/pharmaceutics14061157

**Published:** 2022-05-28

**Authors:** Myongjae Lee, Yoo-Seong Jeong, Min-Soo Kim, Kyung-Mi An, Suk-Jae Chung

**Affiliations:** 1 College of Pharmacy, Seoul National University, Seoul 08826, Korea; myislet@snu.ac.kr (M.L.); jus2401@snu.ac.kr (Y.-S.J.); misol@snu.ac.kr (M.-S.K.); 2 Ildong Pharmaceutical Co., Ltd., Hwaseong 18449, Korea; kman@ildong.com; 3 Research Institute of Pharmaceutical Sciences, Seoul National University, Seoul 08826, Korea

**Keywords:** IDP-73152, human scaling, physiologically-based pharmacokinetic modeling, peptide deformylase inhibitor

## Abstract

IDP-73152, a novel peptide deformylase inhibitor with an antibacterial effect against Gram-positive bacteria, is in phase I development. The objective of this study was to develop a physiologically-based pharmacokinetic model (PBPK) for IDP-73152 in animals, and to extend the model to humans. Biopharmaceutical properties of IDP-73152 are determined using in vitro/in vivo experimentations for the PBPK model. A transit model consisting of gastrointestinal segments is applied for an estimation of the intestinal absorption kinetics. The PBPK model of IDP-73152 in rats is able to appropriately predict the plasma concentration–time profiles after the administration of IDP-73152 at different doses and by different routes (combined absolute average fold error (cAAFE), 1.77). The model is also found to be adequate in predicting the plasma concentration–time profiles of IDP-73152 in mice (cAAFE 1.59) and dogs (cAAFE 1.42). Assuming the oral administration of IDP-73152 to humans at doses of 640 and 1280 mg, the model is able to reproduce the concentration–time profiles obtained in humans (cAAFE 1.38); therefore, these observations indicate that the PBPK model used for IDP-73152 is applicable to animal species and humans. This model may be useful in predicting efficacious doses of IDP-73152 for the management of infectious disease in humans.

## 1. Introduction

Peptide deformylase (PDF), a highly conserved protein in bacteria, catalyzes the removal of N-formyl groups from newly synthesized polypeptides [1,2,3]. Since PDF plays a critical role in the survival of micro-organisms, despite the function of the PDF homolog being apparently unnecessary in mammalian cells [4], the inhibition of this enzyme has been recognized as a promising means for the discovery of new antibiotics. In fact, a considerable number of PDF inhibitors have been previously identified based on their in vitro and in vivo pharmacological activities (e.g., direct bacterio-static/-cidal effects as well as indirect proinflammatory effects), although these inhibitors have been reported to have a number of technical issues, including pharmacokinetic (PK) insufficiencies [5]. For example, actinonin, a natural PDF inhibitor produced by actinomycetes has been found to possess a broad spectrum of antibiotic activity against a number of Gram-positive/-negative strains in vitro [6]; however, this compound and its derivatives have been found to be ineffective in vivo, apparently due to their poor intestinal absorption in animals [7]. BB-83698, another PDF inhibitor, has also been reported to have a problematic oral bioavailability in humans, despite the fact that BB83698 possesses an improved efficacy compared with that of actinonin [8]. Thus far, no antibiotic agent based on PDF inhibition has been commercially developed.

IDP-73152 is an aminopiperidine derivative and a novel inhibitor of the bacterial PDF [9,10]. The inhibitor is currently the subject of a phase I study in Korea for use in the treatment of complicated skin and respiratory infections. This investigational new drug has been demonstrated to apparently have adequate PK properties without clinically significant adverse effects when a single oral dose of 40–1280 mg is given to human subjects [11]. In a previous study [9], it was found that the oral dose at which the survival rate was 50% (ED_50_), based on twice-a-day dosing, was generally less than 20 mg/kg in infected mice models (i.e., mice infected with penicillin-resistant *Streptococcus pneumonia* (the ED_50_ of 3.0 mg/kg), vancomycin-resistant *Enterococcus faecium* (the ED_50_ of 13.3 mg/kg), or methicilin-resistant *Staphylococus aureus* (the ED_50_ of 16.6 mg/kg)). Furthermore, in a preliminary screening study, the in vitro minimum inhibitory concentration values of the compound against the bacterial strains were comparable to, or less than, those of linezolid and vancomycin. Despite these favorable data for the compound, however, the efficacious dose has to be rationally determined based on PK/PD relationships in humans. Unfortunately, the PK characteristics (e.g., PK model structure and parameters) have not yet been systematically determined for IDP-73152 in the literature.

The primary objective of this study was to develop a PK model for IDP-73152 in humans. In particular, we intended to construct/validate a bottom-up physiologically-based pharmacokinetic (PBPK) model of the compound in animals, and to determine whether the model could be extended to humans. We herein report that a PBPK model for IDP-73152, appropriate for three animal species, was constructed and is able to adequately predict the PK in humans.

## 2. Materials and Methods

### 2.1. Materials

**Figure 1:**
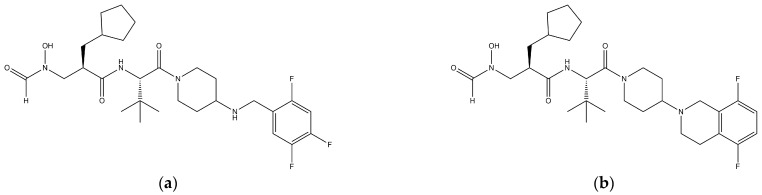
Chemical structure of (**a**) IDP-73152 (C28H41F3N4O4, Mw of 554) and (**b**) internal standard (i.e., IDP-117293; C30H44F2N4O4, Mw of 562).

IDP-73152 (98.5% purity, mesylated salt form, Figure 1a), and the internal standard (i.e., IDP-117293, Figure 1b), were provided by Ildong Pharmaceutical Co., Ltd. (Seoul, Korea). N-2-hydroxyethylpiperazine-N-2-ethane sulfonic acid (HEPES) and Hank’s balanced salt solution (HBSS) were purchased from ThermoFisher Scientific (Waltham, MA, USA) and Sigma-Aldrich (St. Louis, MO, USA). Blank plasma samples were obtained from Biomedex (Seoul, Korea). Liver microsomes from rats, mice, dogs, and humans (Corning^®^ Gentest), and a NADPH-regenerating system, were purchased from Corning Inc. (Glendale, AZ, USA). Williams’ media E, fetal bovine serum, penicillin-streptomycin, and an insulin-transferrin-selenium liquid media supplement were obtained from Sigma-Aldrich^®^ (Merck KgaA, Darmstadt, Germany). Acetonitrile, methanol, water, and formic acid, in HPLC or LC-MS grade, were purchased from Honeywell Research Chemicals (Charlotte, NC, USA). The reagents were used without further purification.

### 2.2. In Vitro PK Studies

#### 2.2.1. Caco-2 Cell Permeability

In this study, bidirectional permeability assays were conducted as described in the literature [12], with minor modifications. Briefly, Caco-2 cells (KCLB, Seoul, Korea) were seeded on Transwell^®^ inserts (Corning Inc., Glendale, AZ, USA) and grown in a humidified atmosphere of 5% CO_2_ at 37 °C to form a confluent monolayer over 3 weeks. On day 21, after seeding, the integrity of the cell monolayer was assessed by measuring the transepithelial electrical resistance using an epithelial voltohmmeter (EVOM and EVOMX, World Precision Instruments, Sarasota, FL, USA). The medium in donor and receiver compartments was then replaced with 5 mM of HEPES in HBSS, and preincubated for 10 min at 37 °C. The permeability study was initiated by adding IDP-73152 (in a 10 µM final concentration in the chambers) to an apical insert (for apical-to-basolateral (A-to-B) transport) or a basolateral chamber (for basolateral-to-apical (B-to-A) transport). Samples were collected from both compartments at 30 min intervals up to 2 h. Throughout the study, the temperature and atmosphere of the compartments were maintained at 37 °C and 5% CO_2_. The samples were stored at −80 °C until analysis and the concentration of the compound was determined by LC-MS/MS assay for IDP-73152 [10]. The apparent permeability coefficient (*P_app_*, in cm/s) was calculated using the following equation:

(1) $$P_{app}=\left(\frac{dQ/dt}{C_{0}\times A}\right)$$

where *dQ*/*dt* is the rate of IDP-73152 transport across monolayers (nmol/s), *C*_0_ is the initial concentration of IDP-73152 (10 μM), and *A* is the surface area of the insert (1.12 cm^2^). The efflux ratio of IDP-73152 in the Caco-2 cell system was calculated by the ratio of *P_app_* from B-to-A transport (*P_app,B-to-A_*) to *P_app_* from A-to-B transport (*P_app,A-to-B_*).

#### 2.2.2. Free Faction of IDP-73152 in the Plasma, Microsomal and Hepatocyte Incubation

The unbound fractions of IDP-73152 in various matrices were measured according to the manufacturer’s instructions for the rapid equilibrium dialysis (RED) device (ThermoFisher Scientific, Waltham, MA, USA) [13]. Briefly, IDP-73152 was added to the plasma or the microsomal/hepatocyte incubation buffer from various species (i.e., mouse, rat, dog, and human for microsomal incubation; rat for hepatocyte incubation) to obtain final concentrations of the compound at 0.3, 1, and 3 μM. After the membrane insert was placed in the RED plate, the solution containing IDP-73152 was added (400 µL) to the sample chamber, and the dialysis buffer (600 μL) was added to the buffer chamber. The RED plate was sealed with parafilm and then incubated at 37 °C in the orbital shaker (Lab Companion, Daejeon, Korea) at 100 rpm for 5 h. After a 50 μL aliquot was aspirated from both the sample and buffer chambers, an equal volume of protein-free buffer or blank medium was replaced in the aspirated sample or buffer to render the matrices compositionally identical. The IDP-73152 concentration in the buffer/sample chambers (i.e., C_Buffer Chamber_ and C_Sample Chamber_) was determined by an assay [10]. Thus, the fraction unbound was defined as:

(2) $$\%\;\mathrm{Fraction}\;\mathrm{unbound}=\left(\frac{C_{\mathrm{Buffer}\;\mathrm{Chamber}}}{C_{\mathrm{Sample}\;\mathrm{Chamber}}}\right)\times 100\%$$

When necessary, the stability of IDP-73152 in the matrix was also determined at 5 h.

#### 2.2.3. Blood Partitioning

In this study, the partitioning of IDP-73152 in blood samples obtained from mice, rats, dogs, and humans was determined using the LC-MS/MS-based depletion method [14] with slight modifications. DP-73152 was added to an aliquot (1 mL) of blank blood or plasma at a final concentration of 0.3, 1, or 3 μM. The mixture was incubated at 37 °C in a water bath for 60 min. After the incubation, the plasma was separated by centrifugation of the blood samples for 5 min at 10,000× *g*/4 °C and stored at −80 °C until analysis [10]. The blood-to-plasma concentration ratio (*BP*) was calculated using the equation:

(3) $$BP=C_{PL}^{Ref}/C_{PL},$$

where $C_{PL}^{Ref}$ is the concentration of the compound at the corresponding blood concentration in the plasma, and *C_PL_* is the concentration from the plasma sample separated from the incubated blood.

#### 2.2.4. Metabolic Stability and Blood Stability

In this study, the metabolic stability of IDP-73152 in microsomes from mice, rats, dogs, and humans, as well as rat hepatocytes, was determined. For a microsomal incubation study, the incubation mixtures, containing mouse, rat, dog, or human liver microsomes at a protein concentration of 0.5 mg/mL, 3.3 mM MgCl_2_, and 1.3 mM NADPH, in a 100 mM potassium phosphate buffer (pH 7.4), were preincubated for 3 min at 37 °C. In parallel, a control incubation mixture (i.e., identical composition to that of the incubation mixture without NADPH) was also prepared for comparison. The reaction was initiated by the addition of a 10× solution of IDP-73152 (final concentration in mixtures of 1 μM) to the incubation mixtures or the control incubation mixtures. The mixtures were incubated in a shaking water bath (37 °C, 90 rpm). Aliquots (30 μL) were collected at 0, 15, 30, and 45 min from the mixture and the reaction was terminated by the addition of a 4-fold volume of ice-cold acetonitrile to the sample. The mixture was then vortexed for 5 min and centrifuged for 10 min at 10,000× *g*. The supernatant was collected and stored at −80 °C until the analysis [10].

For the hepatocyte incubation study, rat hepatocytes were freshly prepared using the two-step collagenase perfusion method with slight modifications [15,16,17]. After the isolation, cell viability during the preparation process was first determined by a trypan blue exclusion assay prior to the stability study. The preparation was considered adequate for subsequent studies if the viability was greater than 80%. When appropriate, the hepatocytes were resuspended in Williams’ media E containing 10% fetal bovine serum, 1% penicillin-streptomycin, and 0.01% insulin-transferrin-selenium (i.e., 10 mg/mL insulin, 5.5 mg/mL transferrin, and 5 mg/mL selenium) at pH 7.4. The reaction was carried out with the hepatocyte suspension prepared at 1 × 10^6^ cells/mL (0.5 mL) and initiated by the addition of equal volume of IDP-73152 solution (i.e., final concentration of the compound at 1 μM in 0.5 × 10^6^ cells/mL of hepatocytes) to the hepatocyte suspension. The mixture was agitated in a shaking incubator at 90 rpm/37 °C, and an aliquot (50 μL) was collected at 0, 15, 30, and 60 min after the initiation of the reaction. The reaction in the sample was terminated by the addition of twice the volume of ice-cold acetonitrile, and the mixture was vortexed (1 min)/centrifuged (for 5 min at 10,000× *g*). The supernatant was collected and stored at −80 °C until analysis [10]. Assuming first-order disappearance kinetics, the slope *k* of the logarithmically transformed IDP-73152 concentration in the sample versus time plot was determined by the linear regression analysis of the data. The total intrinsic clearance (*CL_int,mic_*, μL/min/mg protein for microsomal incubation study and *CL_int,hep_*, μL/min/million cells for hepatocytes incubation study) was then calculated using the following equations:

(4) $$CL_{int,\;mic}=k\times \frac{Volume\;of\;incubation\;medium\;\left(\mu L\right)}{Protein\;in\;incubation\;medium\;\left(\mathrm{mg}\right)}$$

(5) $$CL_{int,hep}=k\times \frac{Volume\;of\;incubation\;\left(\mu L\right)}{Number\;of\;cells\;in\;incubation\;\left(\times 10^{6}\right)}$$

The unbound intrinsic clearance, with respect to the whole liver (*CL_u,int,H_*, L/h), was then estimated for the two cases.

For studies with microsomes:

(6) $$CL_{u,int,H}=\frac{CL_{int,\;mic}}{f_{u,mic}}\times MPPGL\times V_{LI}\times \rho \times \frac{60}{1,000,000}$$

For studies with hepatocytes:

(7) $$CL_{u,int,H}=\frac{CL_{int,\;hep}}{f_{u,hep}}\times HPGL\times V_{LI}\times \rho \times \frac{60}{1,000,000},$$

where *f_u,mic_* and *f_u,hep_* represent the unbound fraction of IDP-73152 in the incubation medium containing microsomes and hepatocytes; *MPPGL* is the amount of microsomal protein per gram of liver; *HPGL* is the hepatocellularity per gram of liver; *V_LI_* is the volume of the liver; and ρ is the density of the liver (i.e., 1.07) [18]. In this study, the values of *MPPGL* of 45 and 39.8 mg protein/g liver were used for animals and humans, respectively [19,20]. In addition, the rat *HPGL* of 120 million cells/g liver was used in this study [21].

From a preliminary study, it was found that IDP-73152 was stable in the plasma of mice, dogs, and humans. In contrast, however, the compound was unstable in the rat blood and plasma. Accordingly, the kinetics of IDP-73152 instability were determined in the rat blood at a final concentration of 0.3 μM. The blood containing IDP-73152 was incubated at 37 °C in a water bath, and aliquots (50 μL) were collected from the mixture at 0, 10, 30, 60, 120, and 240 min. The samples were centrifuged for 5 min at 10,000× *g*/4 °C, and the plasma was collected/stored at −80 °C until analysis [10]. For the estimation of the clearance in the blood (*CL_blood_*), in rats, the disappearance of IDP-73152 in the blood was assumed to be mediated by first-order kinetics. The disappearance rate constant (i.e., the slope *k* of the logarithmically transformed IDP-73152 concentration in the sample versus time plot), *k*, was multiplied by the total blood volume (13.1 mL/200 g rat; i.e., $CL_{blood}=k\times total\;volume\;of\;the\;blood\times BP\;$) to calculate *CL_blood_*.

### 2.3. In Vivo PK Studies

The in vivo experimental protocol was approved by the Institutional Animal Care and Use Committee of Research Laboratory at Ildong pharmaceutical Co., Ltd. (Ref. No.: IDL-PDF-001 and 09005-11004). Animals were acclimatized to the laboratory conditions for at least 1 week before the experiments and were kept in the standardized conditions (20–25 °C, 55 ± 5% humidity, and a 12 h light/dark cycle). The animals fasted overnight prior to drug administration, and food was resupplied 4 h post-dose.

#### 2.3.1. PK Studies in Rats

Male Sprague Dawley rats (Orient Bio Inc., Seongnam, Korea), weighing 194.7 g to 205.4 g, were used in this study. IDP-73152 was administered to rats at a single dose of 2.5, 5, or 10 mg/kg via tail vein injection (the injection volume fixed at 2 mL/kg in saline). Separately, a single 20 mg/kg dose of IDP-73152 was administered via oral gavage at the injection volume of 2 mL/kg in distilled water. Blood samples (100 μL) were collected from the jugular vein at 0.167, 0.33, 0.5, 1, 2, 4, and 8 h after the administration. Plasma was separated/collected by centrifugation of the blood samples for 5 min at 10,000× *g*/4 °C and stored at −80 °C until analysis [10]. When necessary, the biliary and renal excretion of IDP-73152 were studied in bile duct-cannulated rats after the intravenous injection (5 mg/kg) to investigate its route(s) of excretion in rats [22]. Bile and urine samples were collected up to 8 h (i.e., approximately 5 times of the terminal phase half-life) after the administration and the samples were stored at −80 °C until the analysis [10]. The cumulative excretion of the bile and the urine to infinite time was determined by assuming that the excretion was practically completed by the last collection time.

In this study, the extent of tissue distribution of IDP-73152 was also determined in rats. Rats received IDP-73152 at a constant rate of infusion (i.e., the rate of 0.8 mg/h (in 0.6 mL/h)) using a syringe pump (PHD ULTRA, Harvard apparatus, Holliston, MA, USA). The steady state was assumed to have been achieved when IDP-73152 was infused for 10 h (see below). Upon completion of the infusion, the animal was sacrificed, and major tissues (i.e., adipose tissue, brain, heart, kidney, liver, lung, muscle, skin, spleen, and testis) were collected. Assuming the density of tissues to be 1 g/mL, tissue samples were homogenized in twice their volume of phosphate buffered saline using a homogenizer (Ultra Turrax homogenizer, IKA-Werke GmbH & Co. KG, Staufen, Germany). Plasma samples and tissue homogenates were stored at −80 °C until analysis [10]. The tissue-to-plasma concentration ratio at steady state (*K_P,ss_*) was then calculated for each tissue using the following equation [23,24,25]:

(8) $$K_{P,ss}=C_{tissue,ss}/C_{plasma,ss},$$

where *C_tissue,ss_* and *C_plasma,ss_* represent the concentration of the compound in the tissue and plasma at steady-state.

#### 2.3.2. PK Studies in Mice and Dogs

In this study, a single dose of IDP-73152 at 10 mg/kg (i.e., the injection volume of 5 mL/kg, IDP-73152 dissolved in saline) was intravenously administered to male ICR mice (body weight 18.2–21.4 g, Orient Bio Inc., Seongnam, Korea) via the tail vein. Separately, a single dose of IDP-73152 at 20 mg/kg was orally administered using an oral gavage to the mouse. Blood samples (100 μL) were collected from the retroorbital plexus at 0.167, 0.333, 0.5, 1, 2, 4, or 8 h after the administration. Plasma was separated/collected from the blood samples by centrifugation for 5 min at 10,000× *g*/4 °C and stored at −80 °C until analysis [10].

Male beagle dogs (body weight 9.55–10.3 kg, Beijing Marshall Biotechnology, Beijing, China) received a single intravenous dose of 10 mg/kg IDP-73152 via the cephalic vein (i.e., the injection volume of 1 mL/kg, IDP-73152 in saline). When necessary, a single dose of IDP-73152 at 20 mg/kg was also orally administered to dogs in the form of hard gelatin capsules. Blood samples were collected from the jugular vein at 0.167, 0.33, 0.5, 1, 2, 4, and 8 h (for intravenous administration study), and 0.167, 0.33, 0.5, 1, 2, 4, 6, and 8 h (for oral administration study) after the administration. Plasma was separated/collected from the blood sample by centrifugation for 5 min at 10,000× *g*/4 °C and stored at −80 °C until the analysis [10].

#### 2.3.3. Human Study

The PK data for the single-dose administration in humans were obtained from a previous clinical study (ClinicalTrials.gov registry number: NCT01904318) [11]. Briefly, blood samples were collected at 0.25, 0.5, 0.75, 1, 1.5, 2, 2.5, 3, 4, 6, 8, 10, 12, 24, 36, and 48 h after a single oral administration of 640 mg and 1280 mg of IDP-73152. Plasma was separated/collected from blood samples by centrifugation for 5 min at 10,000× *g*/4 °C and stored at −80 °C until the analysis [10].

### 2.4. PBPK Modeling of IDP-73152

#### 2.4.1. Model Structure

**Figure 2:**
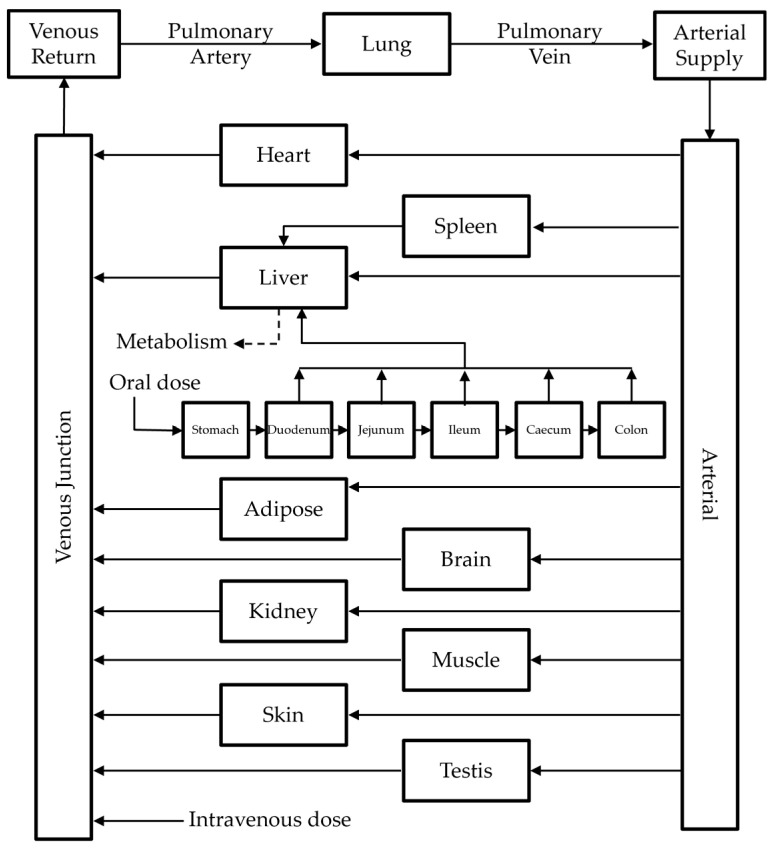
PBPK model structure applied for the IDP-73152 PK in mice, rats, dogs, and humans.

In this study, the PBPK model involving ten tissues (i.e., adipose tissue, brain, heart, kidney, liver, lung, muscle, skin, spleen, and testis (Figure 2; see Appendix A for detailed mathematical descriptions)) was considered to be applicable for the description of IDP-73152 PK in rats, mice, dogs, and humans. Physiological variables were obtained from the literature [26,27,28]; they are summarized in Supplementary Table S1.

#### 2.4.2. Model Development

Since the recovery of intact IDP-73152 in the urine and bile was found to be negligible (i.e., 0.512 ± 0.154% of the dose for biliary recovery and 1.21 ± 0.48% of the dose for urinary recovery) after the intravenous administration of 5 mg/kg of IDP-73152 to rats, the urinary and biliary excretion were assumed to be kinetically insignificant for the compound in rats. In addition, from metabolic stability studies, it was evident that IDP-73152 was metabolically unstable in the incubations containing liver microsomes (from all species)/hepatocytes (from rats) and in the rat plasma. In contrast, IDP-73152 was found to be stable in the plasma from mice, dogs, and humans. Accordingly, during model development, we initially assumed that IDP-73152 was entirely eliminated by the metabolism in the liver (namely, *CL_h_*) and in the blood (namely, *CL_blood_*) in rats: For other species (i.e., mice, dogs, and humans), the elimination was assumed to be primarily mediated by metabolism in the liver. From the results of in vitro metabolic stability studies, the hepatic clearance (*CL_h_*) in rats was estimated under the assumption that the well-stirred liver model was adequate for IDP-73152 [29]:

(9) $$CL_{h}=\frac{Q_{LI}\times BP\times f_{u,p}\times CL_{u,int,H}}{Q_{LI}\times BP+f_{u,p}\times CL_{u,int,H}},$$

where *Q_LI_*, and *f_u,p_* represent the liver blood flow and the unbound fraction of IDP-73152 in the plasma, respectively.

For the estimation of the volume of the distribution of IDP-73512 in rats, the steady-state volume of distribution (*V_ss_*) was calculated using the following equation [30]:

(10) $$V_{ss}=V_{P}+V_{rbc}\times EP+\sum V_{T,i}\times K_{P,ss,i},$$

where *V_p_*, *V_rbc_*, and *V_T,i_* are the volumes of plasma, red blood cells, and tissues, respectively. The erythrocyte-to-plasma partition coefficient (*EP*), is calculated by $EP=1+\frac{\left(BP-1\right)}{Hematocrit}$. The *K_P,ss_* for each tissue obtained from Equation (8) was applied for Equation (10). For the case of non-eliminating organs, the *K_P,ss_* was regarded as the tissue-to-plasma partition coefficient (*K_P_*) in PBPK equations (Appendix A). For the case of the liver—i.e., the eliminating organ—the *K_P,ss_* (*K_P,ss,LI_*) had to be corrected to account for the equilibrium being altered by the elimination. Thus, a PBPK-operative *K_P_* for liver (*K_P,LI_*) was calculated using the following equation [31]:

(11) $$K_{P,LI}=K_{P,ss,LI}/\left(1-ER\right),$$

where *ER* is the hepatic extraction ratio of the compound (i.e., *CL_h_*/*(Q_LI_·BP)*). In this study, a compartmental absorption and transit (CAT) model consisting of stomach and five intestinal segments was considered [32] for the description of the intestinal absorption kinetics of IDP-73152 in animals and humans. We assumed that the initial compartment denoted the stomach (Equation (12)), and the subsequent three compartments represented the small intestine and two additional compartments for the large intestine (i.e., a total of six compartments, with absorption occurring in the five intestinal compartments) (Equation (13)). The differential equations describing the rate of drug absorption and transit in the gastrointestinal tract can be written as:

For the stomach:

(12) $$\frac{dM_{s}}{dt}=-K_{s}\times M_{s}$$

For the intestine, *viz. i* = 1~5:

(13) $$\frac{dM_{i}}{dt}=K_{t,i-1}\times M_{i-1}-(K_{t,i}+k_{a,i})\times M_{i},$$

where *M_s_* is the amount of drug in the stomach; *M_i_* is the amount of drug in the *i*-th intestinal segment; *K_s_*, *K_t,i_*, and *k_a,i_* are the rate constants of gastric emptying, intestinal transit, and the segmental absorption rate, respectively. In Equation (13) at *i* = 1, *K_t,_*_0_ and *M*_0_ represent *K_s_* and *M_s_*, respectively. In this study, the *K_s_* and *K_t,i_* values were obtained from the literature [33,34] (Supplementary Table S2) and used for the PBPK calculations. The absorption rate constant in the intestinal segments in humans, *k_a,i,human_*, was assumed to be identical along the segments and calculated as follows: first, *P_eff_* was empirically estimated from the Caco-2 cell permeability (*P_app_*) [35,36]:

(14) $$LogP_{eff}=0.4926\times LogP_{app}-0.1454$$

Then, *k_a,i,human_* for the intestinal segments *i* = 1~5 was calculated by

(15) $$k_{a,1\sim 5,human}=2\times P_{eff}/R$$

In this formula, *R* represents the radius of the intestinal segment (1.53 cm) in humans [37]. The absorption rate constants in animal species (*k_a,i,rodent_* and *k_a,i,dog_*) were assumed to be estimated by the multiplication of the *k_a,i,human_* with an inter-species scaling factor (*SF*) for the first three intestinal segments (*i* = 1~3) in rodents (see below), or for all five segments (*i* = 1~5) in dogs:

For rodents,

(16) $$k_{a,1\sim 3,rodent}=k_{a,\;1\sim 3,human}\times SF=2\times P_{eff}/R\times SF$$

For dogs,

(17) $$k_{a,1\sim 5,dog}=k_{a,\;1\sim 5,human}\times SF=2\times P_{eff}/R\times SF$$

In this study, flip-flop kinetics for IDP-73152 were apparently present in mice and rats, whereas this complication was not found in dogs and humans; therefore, considering the potential changes in the absorption rates along the gastrointestinal tract (e.g., differences in the absorption surface area, pH, and distribution of transporters) [38,39], an absorption scale factor, *ASF,* was considered for rodents:

(18) $$\;k_{a,4\sim 5,rodent}=k_{a,\;1\sim 3,rodent}\times ASF=2\times P_{eff}/R\times SF\times ASF,$$

where *k_a,4,rodent_* and *k_a,5,rodent_* are the absorption rate constants in the large intestine of rodents. *ASF* was not considered for dogs and humans. In this modelling study, *SF*, and *ASF*, which best described the concentration–time profiles, were determined using nonlinear regression analyses (WinNonlin software (Version 5.0.1; Pharsight, Mountain View, CA, USA)).

#### 2.4.3. Model Extension to Mice and Dogs

In this study, the PBPK model constructed in rats was examined to determine whether the model could be used to predict the PK in mice and dogs. The species-specific input parameters were replaced with the corresponding values for the two species (Table S1). In particular, *CL_blood_* was not considered in mice and dogs, since the plasma instability was virtually absent for IDP-73152 in the plasma. For the distribution kinetics of IDP-73152, a species difference was observed among mice, rats, and dogs; therefore, for the prediction of the *K_P,ss_* values of tissues in difference animal species, the *K_P,ss_*/*f_u,p_* was assumed to be consistent among the three species. *K_p,ss_* values in mice and dogs were then calculated by multiplying the *K_P,ss_*/*f_u,p_* of the rat and *f_u,p_* value for mice or dogs. For the estimation of absorption kinetic parameters, the CAT model was considered as described above (Table S2).

#### 2.4.4. Model Extension to Humans

In this study, the PBPK model established for IDP-73152 in animals was further examined to determine whether the model could be extended to predicting PK in humans. Similar to the model extension study performed for mice and dogs, the CAT model for IDP-73152 was applied for the description of the absorption kinetics in humans. The physiological values, along with gastrointestinal transit times specific for humans, are summarized in Supplementary Tables S1 and S2. To estimate the volume of distribution of IDP-73152 in humans, the *K_P,ss_* values of tissues in humans were estimated by multiplying the *K_P,ss_*/*f_u,p_* of rats with the human *f_u,p_* value.

#### 2.4.5. Determination for the Adequacy of PBPK Model

To evaluate the performance of the PBPK model, the predicted data (*C_predicted_*) were compared to the corresponding mean observed data (*C_observed_*) by calculating the fold error and absolute average fold error (*AAFE*) as follows [40]:

(19) $$fold-error=\frac{C_{predicted}}{C_{observed}}$$

(20) $$AAFE=10^{\frac{\sum \left|\log\left(fold-error\right)\right|}{N}},$$

where *N* is the number of observations.

The model performance was also assessed using the percentage of outliers falling outside the preselected fold-error ranges of [0.5–2.0] or [0.33–3.0]. The predictions were considered acceptable if the percentages of outliers falling outside the fold-error ranges of [0.5–2] and [0.33–3] decreased to below 30% and 20%, respectively. *AAFE* values of 1.9 or less were considered to indicate acceptable model performance [40].

### 2.5. Data and PK Analysis

In this study, data were expressed as mean ± standard deviation (SD), except when such expression was not possible (e.g., parameter estimates from nonlinear regression analysis). When it was necessary to compare mean values, one-way analysis of variance (ANOVA) was used.

When necessary for a model independent PK analysis, a standard moment analysis was carried out with WinNonlin software using either representative (for the study with mice) or individual (for the study with rats, dogs, and humans) concentration–time profile(s). For the calculation of PBPK models, Berkeley Madonna 8.3.18 (Albany, CA, USA) was used with the fourth-order Runge–Kutta method for numerical integration.

## 3. Results

### 3.1. In Vitro PK Studies

#### 3.1.1. Caco-2 Permeability

In this study, the transepithelial electrical resistance values ranged from 251 to 311 Ω∙cm^2^, suggesting that the development of the tight junction was adequate for the monolayers [41]. The Caco-2 cell permeabilities of IDP-73152 were 31.2 ± 1.93 × 10^−6^ cm/s (A-to-B) and 49.5 ± 3.5 × 10^−6^ cm/s (B-to-A), which were higher than those of a high-permeability control (i.e., metoprolol), and is indicative of good permeability across the intestinal barrier for the compound. In particular, the efflux ratio of IDP-73152 was less than 2 in the Caco-2 cell monolayers. The recoveries were 96.6 ± 5.1% and 93.4 ± 3.0% for the A to B transport and B to A transports, respectively.

#### 3.1.2. Protein Binding and Blood Partitioning

The mean *f_u,p_* was apparently comparable amongst the species studied (i.e., 0.0562 ± 0.0087 (mice), 0.0582 ± 0.0050 (rats), 0.0547 ± 0.0053 (dogs), and 0.0744 ± 0.0019 (humans)) for IDP-73152. The percentage of the drug remaining in the plasma was close to 100% in animal species/humans, except for rats (e.g., the percentage remaining at 5 h, 92.3 ± 2.7% (mice), 65.3 ± 13.7% (rats), 101 ± 6% (dogs), and 98.2 ± 3.4% (humans)), which is indicative of a species difference in plasma stability. The *BP* of IDP-73152 was 1.31 ± 0.13 (mice), 1.56 ± 0.37 (rats), 1.45 ± 0.11 (dogs), and 1.24 ± 0.08 (humans). In addition, the *f_u,mic_* of IDP-73152 appeared to be similar amongst the species (i.e., 0.571 ± 0.023 (mice), 0.439 ± 0.043 (rats), 0.507 ± 0.040 (dogs), and 0.579 ± 0.006 (humans)). The *f_u,hep_* of IDP-73152 was found to be 0.492 ± 0.068 in rats.

#### 3.1.3. Estimation of Hepatic Clearance from In Vitro Metabolic Stability Assays

In this study, the metabolic stability of IDP-73152 was determined with the liver microsomes from the three animal species and humans, as well as with isolated rat hepatocytes. From the liver microsomes, *CL_int,mic_* (μL/min/mg protein) was calculated to be 80.1 ± 1.7 (mice), 72.0 ± 2.6 (rats), 62.6 ± 2.4 (dogs), and 31.8 ± 2.2 (humans). When scaled up to the whole liver, the *CL_u,int,H_* values (L/h) were 0.539 ± 0.011 (mice), 7.46 ± 0.27 (rats), 108 ± 4 (dogs), and 206 ± 14 (humans). From the metabolic stability study conducted with rat hepatocytes, *CL_,int,hep_* and *CL_u,int,H_* were estimated to be 27.7 ± 3.2 μL/min/million cells and 6.82 ± 0.79 L/h for rats, respectively. It was noted that the estimated *CL_u,int,H_* values from rat liver microsomes and rat hepatocytes were almost comparable, suggesting that the *CL_h_* can be estimated by either of the two experimental systems. Since we intended to develop a kinetic model for the compound in other animal species and humans, we chose to use the metabolic clearance estimated from a microsomal incubation study as a reference. Based on the hepatic *ER* calculated for IDP-73152, this compound may be categorized as a low-clearance drug (i.e., *ER* of 0.102 ± 0.002 (mice), 0.222 ± 0.006 (rats), 0.106 ± 0.004 (dogs), and 0.159 ± 0.009 (humans)) [42,43].

In a separate study, the metabolic stability of 1 μM of IDP-73152 was studied in 1 mL of fresh whole blood from rats. The percentage of IDP-73152 remaining in incubation declined exponentially with time; the *CL_blood_* was calculated with a percentage of the drug at 0.0430 ± 0.023 L/h/kg. It was found that the in vivo systemic clearance from the plasma (*CL_p_*) (see below; Table 1) was 2.00 ± 0.16 L/h/kg in rats, suggesting that the contribution of *CL_blood_* to the *CL_p_* is minor (2.15%) in rats. For the case of *CL_blood_* in other species, the metabolic stability in the plasma was even lower in mice, dogs, and humans than in rats; therefore, the contribution of *CL_blood_* was assumed to be kinetically insignificant and considered absent in the subsequent modelling study.

### 3.2. In Vivo PK Studies in Preclinical Species

#### 3.2.1. PK Characteristics of IDP-73152 in Preclinical Species

The PK of IDP-73152 was studied in mice, rats, and dogs; a model independent analysis of the data is summarized in Table 1. In general, IDP-73152 had low to moderate *CL_p_* values (from 0.664 to 2.00 L/h/kg in the animal species), moderate volumes of distribution (from 1.15 to 2.54 L/kg), and terminal elimination half-lives between 0.731 and 1.18 h. When administered orally, the compound had a T_max_ of 0.333, 0.667, and 0.777 h for mice, rats, and dogs, respectively. The absolute oral bioavailability of the compound in solution for rodents and capsule for dogs ranged from 55.3% to 95.5%, indicating that the absorption of IDP-73152 in the gastrointestinal tract was rapid and the extent of absorption was over 50%, which is consistent with the findings of in vitro cell permeability and metabolic stability studies.

After an intravenous injection to rats at doses of 2.5, 5, and 10 mg/kg, the key PK parameters of IDP-74152 were not found to be statistically different, as evidenced by the consistent *CL_p_* (1.85–2.00 L/h/kg) and *V_ss_* (2.45–2.78 L/kg). As neither *CL_p_* nor *V_ss_* were statistically different amongst the doses (one-way ANOVA), linear PK were assumed for IDP-73152 in the kinetic modelling study.

#### 3.2.2. Tissue Distribution of IDP-73152

Considering the *CL_p_* in rats of approximately 2.00 ± 0.16 L/h/kg at 10 mg/kg (Table 1), the expected steady-state concentration was 2.12 ± 0.34 μg/mL when the compound was intravenously infused at a rate of 0.8 mg/h (i.e., 0.6 mL/h; 1.33 mg/mL of IDP-73152 in citrate buffer pH 6.0) for 10 h. The experimental concentrations after 8 h of infusion (in µg/mL, 2.07 ± 0.36 (8 h), 1.91 ± 0.27 (9 h), and 2.12 ± 0.34 (10 h)) were not found to be statistically different from each other (one-way ANOVA). These observations suggest that the steady-state plasma concentration is already achieved for the drug by 8 h in rats. Accordingly, a 10 h infusion study was carried out in rats for the determination of the *K_P,ss_*. In general, the concentration of IDP-73152 in highly perfused organs, except for the brain, was found to be higher than that in the plasma (Table 2). As a result, the *V_ss_* calculated by the Equation (10), was estimated to be 2.09 L/kg, which was comparable to the value obtained from the moment analysis (i.e., 2.54 ± 0.26 L/kg); therefore, the ten-tissue model was considered to be adequate for the prediction of the PK of IDP-73152 in rats.

### 3.3. PBPK Modeling

#### 3.3.1. Model Development and Comparison with Experimental Data for IDP-73152

**Figure 3:**
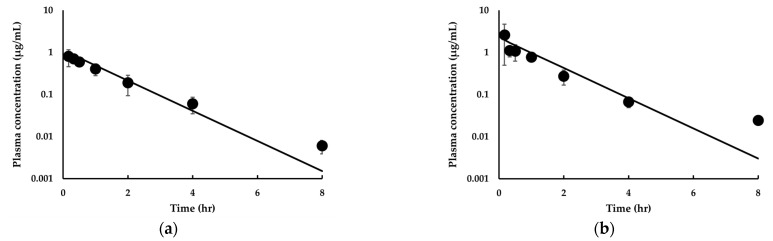

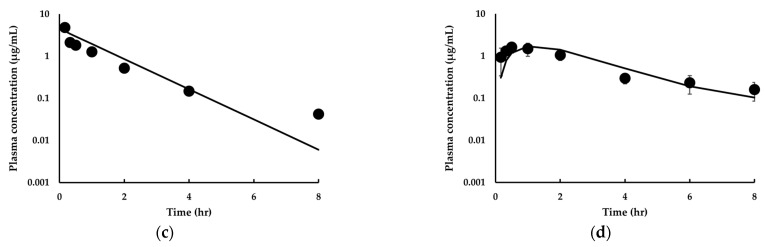
Observed and predicted time-concentration profiles for IDP-73152 following (**a**) 2.5 mg/kg, (**b**) 5 mg/kg, (**c**) 10 mg/kg intravenous, and (**d**) 20 mg/kg oral administration in rats. Solid lines represent predicted data. Closed circles (•) represent observed data. Observed data means ± SD (*n* = 3 rats for intravenous administration study, *n* = 6 rats for oral administration study).

In this study, we attempted to construct a PBPK model for IDP-73152 in rats, primarily using biopharmaceutical data obtained for rats. When the plasma concentration–time profiles for IDP-73152 were predicted with the model assuming a single intravenous dose of 2.5, 5, or 10 mg/kg to rats, the model prediction was apparently comparable to the experimental concentration–time profiles (Figure 3a–c). The percentage of outliers falling out of the 2-fold, 3-fold, and *AAFE* was 14.3%, 14.3%, and 1.86, respectively, for the intravenous administration to rats. Using the data of 20 mg/kg oral administration of IDP-73152 to rats (Figure 3d), the *SF* and *ASF* were fitted to be 0.297 and 0.0568, respectively. The percentage of outliers falling out of the 2-fold, 3-fold, and *AAFE* was 12.5%, 12.5%, and 1.56, respectively, for the case of rat oral administration. As a result, combined *AAFE* (cAAFE) for the intravenous and oral administration of IDP-73152 was 1.77 in rats. In addition, the observed C_max_ and AUC_inf_ values (Table 3) after oral administration of IDP-73152 in rats were consistent with the model calculations (i.e., 1.02-fold and 1.03-fold, respectively). These observations indicate that the current PBPK model was able to adequately predict the plasma concentration–time profiles of IDP-73152 in rats.

#### 3.3.2. Model Extension to Mice and Dogs for IDP-73152

**Figure 4:**
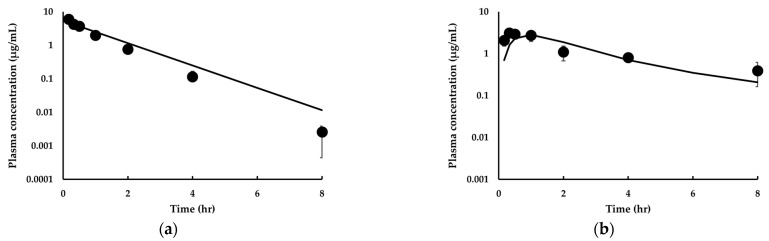
Observed and predicted time-concentration profiles for IDP-73152 following (**a**) 10 mg/kg intravenous and (**b**) 20 mg/kg oral administration in mice. Solid lines represent predicted data. Closed circles (•) represent observed data. Observed data are means ± SD (*n* = 3 mice for intravenous administration study, *n* = 4 mice for oral administration study).

To evaluate whether the PBPK model was also applicable to mice and dogs in predicting IDP-73152 PK, simulations were conducted. In this study, *SF* values of 0.446 and 1.31 for mice and dogs, respectively, were estimated, whereas the *ASF* for mice was 0.0973. The model prediction for the mouse PK profile is presented in Figure 4. The percentages of outliers falling outside the 2-fold, 3-fold, and *AAFE* were 28.6%, 14.3%, and 1.57, respectively, for the intravenous administration of the compound to mice (Figure 4a). In addition, the percentages of outliers falling outside the 2-fold, 3-fold, and *AAFE* were 14.3%, 0.00%, and 1.60, respectively, for the oral administration of the drug to mice (Figure 4b). As a result, the cAAFE of IDP-73152 was 1.59 in mice. In addition, the observed C_max_ and AUC_inf_ values (Table 4) after oral administration of IDP-73152 in mice were consistent with the model calculations (i.e., 0.906-fold and 0.880-fold, respectively).

**Figure 5:**
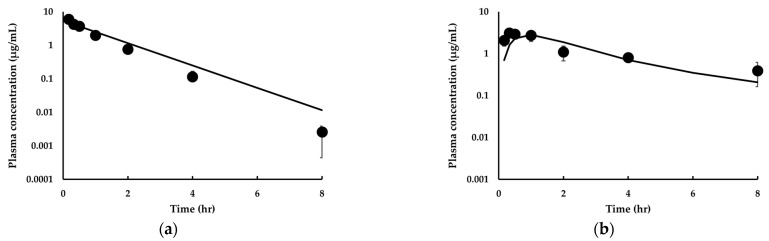
Observed and predicted time–concentration profiles for IDP-73152 following (**a**) 10 mg/kg intravenous and (**b**) 20 mg/kg oral administration in dogs. Solid lines represent predicted data. Closed circles (•) represent observed data. Observed data are means ± SD (*n* = 3 dogs).

The predicted dog PK profiles are also shown in Figure 5. The percentages of outliers falling outside the 2-fold, 3-fold, and *AAFE* were 12.5%, 12.5%, and 1.44, respectively, for the intravenous administration of the compound to dogs (Figure 5a). In addition, the percentages of outliers falling outside the 2-fold, 3-fold, and *AAFE* were 12.5%, 0.00%, and 1.27, respectively, for the oral administration of the drug to dogs (Figure 5b). As a result, the cAAFE of IDP-73152 was 1.42 in dogs. In addition, the observed C_max_ and AUC_inf_ values (Table 5) after oral administration of IDP-73152 in dogs were consistent with the model calculations (i.e., 0.832-fold and 1.08-fold, respectively). These observations collectively suggest that the PBPK models are adequate for use in reproducing the plasma concentration–time profile of IDP-73152 in mice and dogs.

#### 3.3.3. Estimation of Human PK for IDP-73152

**Figure 6:**
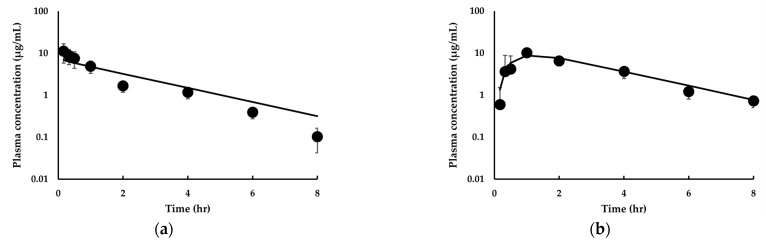
Human PBPK model prediction of plasma IDP-73152 concentrations following (**a**) 640 and (**b**) 1280 mg oral administration in humans. Solid lines represent predicted data. Closed circles (•) represent observed data. Observed data are means ± SD (*n* = 8 humans).

Since the current model was apparently adequate for estimating the plasma PK profiles in the three animal species, we reasoned that the model could be further extended to describe the kinetics in humans. It was noted that, in a phase I study, the human data were only available in the case of the oral administration of IDP-73152. Similar to the preclinical animal species, the CAT model was considered to be adequate for predicting the kinetics of intestinal absorption of the compound in humans. Under these conditions, the model-predicted PK profiles in humans, assuming a single oral administration of 640 or 1280 mg to fasting humans, are shown in Figure 6 and Table 6. The percentages of outliers falling outside the 2-fold, 3-fold, and *AAFE* were 6.25%, 0.00%, and 1.46, respectively, for a 640 mg administration and 6.25%, 0.00%, and 1.30, respectively, for a 1280 mg administration. As a result, the cAAFE for the two doses of oral administration of IDP-73152 was 1.38 in humans. In addition, the predicted C_max_ and AUC_inf_ values were within 0.498-fold and 1.00-fold, respectively, and the values were obtained from the model independent analyses of the data for a 640 mg oral administration in humans. In addition, the predicted C_max_ and AUC_inf_ values were 0.671-fold and 1.12-fold, respectively, and the values were obtained from model independent analyses of the data for a 1280 mg oral administration in humans [11]. These observations indicate that the current PBPK model for IDP-73152 can be extended to humans for the description of the IDP-73152 PK profile.

## 4. Discussion

Bacterial infections, particularly those caused by penicillin-resistant *Streptococcus pneumonia*, vancomycin-resistant *Enterococcus faecium*, or methicilin-resistant *Staphylococus aureus*, can be serious threats to the general public [44]. In addition, bacteria that are resistant to multiple antibiotic agents can become increasingly problematic [45], especially when there is no other therapeutic option. Considering the fact that the latest discovery of new class of antibiotics occurred in the 1980s, the discovery and development of a novel class of antibiotics is certainly needed. The inhibition of PDF has traditionally been considered as one of such developmental attempts [46,47,48,49]; however, technical issues, including their poor pharmaceutical/PK properties (e.g., low solubility, poor oral absorption), have prevented the commercial development of previous PDF inhibitors [9,50]. IDP-73152 is an aminopiperidine-based PDF inhibitor that is under phase I study in Korea. Based on findings obtained with infected mouse models [9], the compound appears to be potentially useful for the treatment of methicillin-resistant *Staphylococus aureus* and vancomycin-resistant *Enterococcus faecium* infections. In this study, the PK model was developed/validated for IDP-73152 in animal models and was found to be extendable to humans. The effectiveness of the drug may be predicted with the current kinetic model assuming various infectious conditions (e.g., systemic, respiratory tract or skin infections caused bacteria resistant to methicillin, vancomycin, or penicillin).

Initially, the *V_ss_* values were estimated using p*K*a and log *P* values of IDP-73152 (Supplementary Table S3) using the Rodgers and Rowland method [51,52,53]; however, the predicted *V_ss_* value (9.45 L/kg) overestimated the in vivo *V_ss_* obtained rat study (2.45–2.78 L/kg). Thus, in infusion studies carried out with rats, IDP-73152 was found to be readily distributed to highly perfused organs (e.g., liver, heart, lung, and kidney). The drug distribution characteristics in the rat also appeared to be applicable to mice and dogs, as evidenced by the fact that the *V_ss_* values predicted in these animals (i.e., in L/kg 1.98 (mice), 2.09 (rats), 1.58 (dogs)) are consistent with *V_ss_* values calculated by moment analyses (i.e., in L/kg 1.43 (mice), 2.54 ± 0.26 (rats), 1.15 ± 0.46 (dogs)). A similar approach could be applied to predict *V_ss_* in humans (i.e., 1.56 L/kg).

It was found that excretory and *CL_blood_* were negligible for IDP-73152 in rats, suggesting that hepatic metabolism is the major route of elimination for IDP-73152. Consistent with this statement, the in vivo *CL_p_* of the drug (i.e., in L/h/kg, 1.52 (mice), 2.00 ± 0.16 (rats), 0.618 ± 0.013 (dogs)) was almost entirely accounted for by the *CL_h_* calculated from the *CL_int,mic_* of the drug (i.e., in L/h/kg, 1.36 (mice), 1.69 (rats), 0.532 (dogs)). From this estimation, an additional scaling of the intrinsic clearance was considered unnecessary for the estimation of *CL_p_* for the PDF inhibitor. The *CL_p_* was expected to be approximately 0.190 L/h/kg in humans. Our preliminary studies indicate that the primary metabolic pathway is mediated by hepatic CYP3A for IDP-73152. Since the in vivo *CL_p_* was adequately predicted using well-stirred liver model with microsomal stability data, the liver function is likely to have an important factor on the elimination of IDP-73152 (in other words, a low extraction ratio drug). In particular, infection-associated jaundice was reported in 3–25% of pneumonia patients [54], suggesting that those patients may have altered pharmacokinetics for IDP-73152 (e.g., enhanced exposures). Furthermore, an acute kidney injury is a common outcome in sepsis (e.g., with a reported incidence between 15% and 38%): The acute kidney injury may render a reduction in cytochrome P450 activities [55], which would lead to changes in the pharmacokinetics of the PDF inhibitor. In this study, the involvement of drug transporters (e.g., SLC transporters) in IDP-73152 pharmacokinetics was not systematically studied. Although linear pharmacokinetics is likely for the inhibitor (i.e., no dose dependency for *CL_p_* and *V_ss_*; Section 3.2.1) in rats, the dose dependency was not studied for the drug in humans. Furthermore, it was reported that infections altered the activity of transporters [56], and thus, the characteristics of IDP-73152 pharmacokinetics may change during the disease. This aspect of IDP-73152 may warrant additional studies.

IDP-73152 was highly permeable in Caco-2 cell monolayers. The in vitro permeability of IDP-73152 of 31.2 × 10^−6^ cm/sec was higher than that of metoprolol, a high-permeability control. In addition, the compound was unlikely to be a substrate for P-glycoprotein or breast cancer resistance protein [57], as evidenced by its efflux ratio of less than 2 in the Caco-2 cell study. Consistent with the in vitro observations, IDP-73152 had an adequate oral bioavailability in the animal species studied (i.e., 78.1% (mice), 55.3% (rats), 95.5% (dogs)). In particular, the absolute bioavailability from in vivo experiments could be reasonably predicted by theoretical calculations (i.e., 69.2% (mice), 65.4% (rats), 99.9% (dogs)) using the equation of F=Fa×Fg×Fh, where *Fa* is the absorbed amount after oral administration ($Fa=1-\prod \left(\frac{K_{t,i}}{K_{t,i}+k_{a,i}}\right)$) [32], *Fg* is the fraction of a drug passing through the gut wall without metabolism (*Fg* of 1), and *Fh* is the fraction of a drug passing though the liver without metabolism (Fh=(1−ER)). Using a similar approach, the absolute bioavailability in humans was estimated to be 99.9%, indicative of virtually complete oral bioavailability for IDP-73152.

In this study, we found that the *SF* and *ASF* were necessary for the description of the PK after the oral administration of IDP-73152 in animal species. For example, the theoretical bioavailability was calculated to be over 99% in the animals if *SF* was not considered. In addition, the C_max_ ratio, the ratio of C_max_ from the model to that from the observation, was found to deviate significantly (e.g., 1.72 (mice), 2.36 (rats), and 0.760 (dogs)) without the scaling. Furthermore, the *ASF* was also apparently necessary to account for the flip-flop kinetics of the drug in rats and mice (i.e., 0.0568 (rats), 0.0973 (mice)). By applying these scaling factors, the C_max_ ratio and AUC ratio, the ratio of theoretical AUC_inf_ to experimental AUC_inf_, was reduced to 0.880–1.03 in the rodents. To further determine whether *ASF* was necessary, an input rate at each sampling time was estimated by deconvolution (i.e., the area-function method (Supplementary Section S2)) [58]. In this analysis, the rule of superposition was assumed to be applicable (i.e., linear disposition kinetics, Table 3). It was noted that a bi-phasic relationship was evident in the input rate versus time plot for IDP-73152 in the two animal models (Supplementary Figure S1), suggesting that an introduction of *ASF* is valid for the description of IDP-73152 absorption in animals.

PK-PD analysis is likely to be an useful tool to establish the relationship between PK-PD and clinical outcome [59]. In particular, a mechanism-based model, coupled with a pathophysiological PK model, is readily applicable in optimizing the pharmacotherapeutics of antibiotics [60], such as IDP-73152. In this study, the predicted PK profile closely matched that from healthy male volunteers receiving the inhibitor, suggesting that the current PBPK model is adequate in predicting the PK profiles in other population groups (e.g., female volunteers, geriatrics, pediatrics, and patients with infectious disease).

## 5. Conclusions

In conclusion, the PBPK model for IDP-73152 was constructed primarily based on in vitro kinetic/biopharmaceutic properties. The model was able to reproduce the concentration–time profile in the plasma of the drug in at least three animal species and in humans with different doses and routes of administration; therefore, the current PBPK model may be useful for the prediction of PK and an efficacious dose of the PDF inhibitor in various clinical situations.

## Figures and Tables

**Table 1:** IDP-73152 PK parameters following intravenous and oral administration to mice, rats, and dogs.

Parameter		Mice ^1^	Rats ^2^	Dogs ^2^
Intravenous PK			
	Dose (mg/kg)	10	10	10
	*CL_p_* (L/h/kg)	1.52	2.00 ± 0.16	0.664 ± 0.259
	*V_ss_* (L/kg)	1.43	2.54 ± 0.26	1.15 ± 0.46
	AUC_inf_ ^3^ (μg·h/mL)	6.59	5.03 ± 0.38	16.6 ± 5.9
	*t_1_*_/*2*_ (h)	0.731	1.43 ± 0.11	1.18 ± 0.14
Oral PK				
	Dose (mg/kg)	20	20	20
	C_max_ ^4^ (μg/mL)	3.10	1.65 ± 0.44	10.5 ± 2.0
	T_max_ ^5^ (h)	0.333	0.638 ± 0.29	0.777 ± 0.387
	AUC_inf_ (μg·h/mL)	10.9	5.57 ± 1.59	31.6 ± 5.9
	*t_1_*_/*2*_ ^6^ (h)	2.86	2.97 ± 1.40	1.81 ± 0.04
	F ^7^ (%)	78.1	55.3	95.5

^1^ Calculated by representative concentration–time profile. ^2^ Mean ± SD. ^3^ Area under the curve from time of dosing extrapolated to infinity time. ^4^ Maximum drug concentration. ^5^ Time to achieve C_max_. ^6^ Terminal phase half-life. ^7^ Bioavailability.

**Table 2:** Tissue-to-plasma concentration ratio at a steady state of IDP-73152 in rats (*K_P,ss_*).

Tissue	*K_P,ss_* ^1^
Adipose tissue	0.853 ± 0.196
Brain	0.0729 ± 0.0167
Heart	2.15 ± 0.22
Kidney	8.68 ± 1.83
Liver	12.2 ± 3.3
Lung	7.13 ± 1.28
Muscle	1.37 ± 0.30
Skin	1.08 ± 0.38
Spleen	4.82 ± 1.30
Testis	0.338 ± 0.089

^1^ Mean ± SD.

**Table 3:** Observed and predicted PK parameters of IDP-73152 in rats.

Parameter		Value		
Intra venous PK				
	Dose (mg/kg)	2.5	5	10
	Observed AUC_inf_ (μg·h/mL)	1.32 ± 0.13	3.27 ± 1.82	5.03 ± 0.38
	Predicted AUC_inf_ (μg·h/mL)	1.44	2.87	5.74
	AUC ratio ^1^	1.09	0.826	1.14
Oral PK				
	Dose (mg/kg)		20	
	Observed C_max_ (μg/mL)		1.65 ± 0.44	
	Predicted C_max_ (μg/mL)		1.69	
	C_max_ ratio ^2^		1.02	
	Observed AUC_inf_ (μg·h/mL)		5.57 ± 1.59	
	Predicted AUC_inf_ (μg·h/mL)		5.71	
	AUC ratio		1.03	

^1^ AUC ratio = Predicted AUC_inf_/Observed AUC_inf_. ^2^ C_max_ ratio = Predicted C_max_/Observed C_max._

**Table 4:** Observed and predicted PK parameters of IDP-73152 in mice.

Parameter		Value
Intra venous PK		
	Dose (mg/kg)	10
	Observed AUC_inf_ (μg·h/mL)	6.59
	Predicted AUC_inf_ (μg·h/mL)	7.58
	AUC ratio	1.15
Oral PK		
	Dose (mg/kg)	20
	Observed C_max_ (μg/mL)	3.10
	Predicted C_max_ (μg/mL)	2.81
	C_max_ ratio	0.906
	Observed AUC_inf_ (μg·h/mL)	10.3
	Predicted AUC_inf_ (μg·h/mL)	9.06
	AUC ratio	0.880

**Table 5:** Observed and predicted PK parameters of IDP-73152 in dogs.

Parameter		Value
Intra venous PK		
	Dose (mg/kg)	10
	Observed AUC_inf_ (μg·h/mL)	16.6 ± 5.9
	Predicted AUC_inf_ (μg·h/mL)	18.7
	AUC ratio	1.13
Oral PK		
	Dose (mg/kg)	20
	Observed C_max_ (μg/mL)	10.5 ± 2.0
	Predicted C_max_ (μg/mL)	8.74
	C_max_ ratio	0.832
	Observed AUC_inf_ (μg·h/mL)	31.6 ± 5.9
	Predicted AUC_inf_ (μg·h/mL)	34.1
	AUC ratio	1.08

**Table 6:** Observed ^1^ and predicted PK parameters of IDP-73152 in humans.

Parameters	Value	
Dose (mg)	640	1280
Observed C_max_ (μg/mL)	8.92 ± 1.17	13.2 ± 2.50
Predicted C_max_ (μg/mL)	4.44	8.87
C_max_ ratio	0.498	0.671
Observed AUC_inf_ (μg·h/mL)	43.4 ± 4.47	77.4 ± 15.4
Predicted AUC_inf_ (μg·h/mL)	43.4	86.9
AUC ratio	1.00	1.12

^1^ Clinical data from a phase 1 study of IDP-73152 mesylate in healthy male volunteers [11].

## Data Availability

The cited clinical trial data were already published by Shin et al. (NCT01904318) [11].

## Supplementary Materials

The following supporting information can be downloaded at: https://www.mdpi.com/article/10.3390/pharmaceutics14061157/s1, Table S1: Physiological variables [26,27,28]; Table S2: Gastrointestinal transit rate constant [33,34]; Table S3: Physiological properties of IDP-73152. Figure S1: *dF_a(t)_*/*dt*, i.e., the input rate, versu*s* time plots for (a) mice, and (b) rats.

## Appendix A

In this study, a perfusion-limited distribution was assumed to apply for the rate of tissue distribution of IDP-73152 in the given species (e.g., mice, rats, dogs, and humans). Therefore, the rate equation for drug distribution kinetics to typical tissues may be expressed as:

$$V_{T}\frac{dC_{T}}{dt}=Q_{T}\times \left(C_{art,blood}-\frac{C_{T}\times BP}{K_{P}}\right),$$

where *V_T_* is the anatomical volume of the tissue; *C_T_* is the tissue concentration of IDP-73152; *C_art,blood_* is the arterial blood concentration; and $Q_{T}$ is the blood flow to the tissue. For the case of the lung, *C_art,blood_* was replaced with the venous blood concentration (*C_ven,blood_*), while the blood flow was assumed to be the cardiac output.

For the case of the liver, the rate is expressed as:

$$\begin{array}{cc} V_{LI}\frac{dC_{LI}}{dt} & =(Q_{LI}-Q_{SP})\times C_{art,blood}+Q_{SP}\times \frac{C_{SP}\times BP}{K_{P,SP}}-Q_{LI}\times \frac{C_{LI}\times BP}{K_{P,LI}}\\ & \;\;\;\;\;\;\;\;\;\;\;\;\;\;\;\;-CL_{u,int,H}\times C_{LI}\times \frac{f_{up}}{K_{P,LI}}, \end{array}$$

where *C_LI_*, and *C_SP_* are the drug concentrations in the liver, and spleen; *Q_SP_* is the blood flow to the gut and spleen; and *K_P,SP_* is the tissue-to-plasma partition coefficient of the spleen for the drug.

For the venous blood, the rate is written as:

$$V_{ven}\frac{dC_{ven,blood}}{dt}=Q_{AD}\times \frac{C_{AD}\times BP}{K_{P,AD}}+Q_{BR}\times \frac{C_{BR}\times BP}{K_{P,BR}}+Q_{HE}\times \frac{C_{HE}\times BP}{K_{P,HE}}+\;+Q_{KI}\times \frac{C_{KI}\times BP}{K_{P,KI}}+Q_{LI}\times \frac{C_{LI}\times BP}{K_{P,LI}}+Q_{MU}\times \frac{C_{MU}\times BP}{K_{P,MU}}+Q_{SK}\times \frac{C_{SK}\times BP}{K_{P,SK}}+Q_{TE}\times \frac{C_{TE}\times BP}{K_{P,TE}}+\;Q_{RE}\times C_{art,blood}-Q_{LU}\times C_{ven,blood},$$

where *V_ven_* is the volume of venous blood; *Q_AD_*, *Q_BR_*, *Q_HE_*, *Q_KI_*, *Q_MU_*, *Q_SK_*, *Q_TE_*, *Q_LU_*, and *Q_RE_* are the blood flow to the adipose tissue, brain, heart, kidney, muscle, skin, testis, lung, and rest of body; *C_AD_*, *C_BR_*, *C_HE_*, *C_KI_*, *C_MU_*, *C_SK_*, and *C_TE_* are the drug concentrations in the adipose tissue, brain, heart, kidney, muscle, skin, and testis; and *K_P,AD_*, *K_P,BR_*, *K_P,HE_*, *K_P,KI_*, *K_P,MU_*, *K_P,SK_*, and *K_P,TE_* are the tissue-to-plasma partition coefficients of the adipose tissue, brain, heart, kidney, muscle, skin, and testis.

For arterial blood, the rate is expressed as:

$$V_{art}\frac{dC_{art,blood}}{dt}=Q_{CO}\times \left(\frac{C_{LU}\times BP}{K_{P,LU}}-C_{art,blood}\right),$$

where $V_{art}$ is the arterial blood volume.
