# Supplementary material for: Prediction of Pharmacokinetics of IDP-73152 in Humans Using Physiologically-Based Pharmacokinetics
Source: Pharmaceutics. 2022 May 28;14(6):1157. doi: 10.3390/pharmaceutics14061157 (PMC9227536; doi:10.3390/pharmaceutics14061157)
Supplement: Supplementary file 1 [file pharmaceutics-14-01157-s001.zip › pharmaceutics-1736543-supplementary.pdf]

pharmaceutics

Supplementary Material

Prediction of Pharmacokinetics of IDP-73152 in Humans using Physiologically Based Pharmacokinetics

Myongjae Lee[1], Yoo-Seong Jeong[1], Min-Soo Kim[1], Kyung-Mi An[2], Suk-Jae Chung[1,3,*]

[1] College of Pharmacy, Seoul National University, Seoul 08826, Korea; myislet@snu.ac.kr (M.L.); jus2401@snu.ac.kr (Y.-S.J.); misol@snu.ac.kr (M.-S.K.)
[2] Ildong Pharmaceutical Co., Ltd., Hwaseong, Gyeonggi 18449, Korea; kman@ildong.com
[3] Research Institute of Pharmaceutical Sciences, Seoul National University, Seoul 08826, Korea;
* Correspondence: sukjae@snu.ac.kr

Section S1: Physiological parameters

Table S1. Physiological variables [26-28]

Parameter		Mouse	Rat	Dog	Human
Body weight (kg)		0.02	0.2	10	65
Cardiac output (mL/s)		0.192	1.10	34.2	90.05
Lung	%CO	100	100	100	100
	FV	0.00634	0.00810	0.00867	0.0175
Adipose	%CO	1.12	0.835	3.17	6.57
	FV	0.0764	0.0386	0.164	0.274
Muscle	%CO	13.4	15.7	22.6	12.6
	FV	0.369	0.471	0.439	0.350
Liver	%CO	29.5	24.6	28.0	25.0
	FV	0.0665	0.0787	0.0303	0.0226
Spleen	%CO	1.32	1.25	2.26	3.15
	FV	0.00403	0.00232	0.00247	0.00262
Heart	%CO	4.11	8.14	4.89	4.24
	FV	0.00437	0.00463	0.00767	0.00483
Brain	%CO	6.69	2.72	4.70	14.1
	FV	0.0167	0.00478	0.00763	0.0230
Kidney	%CO	18.8	19.2	19.6	22.9
	FV	0.0156	0.0143	0.00500	0.00516
Skin	%CO	8.89	12.1	12.5	5.82

	FV	0.141	0.154	0.0774	0.0403
Testis	%CO	0.435	1.04	0.312	0.170
	FV	0.00592	0.00965	0.00164	0.000674
Venous	FV	0.0452	0.0216	0.0600	0.0637
Arterial	FV	0.0228	0.0436	0.0300	0.0319

Table S2. Gastrointestinal transit rate constant [33,34]

Parameter	Mouse	Rat	Dog	Human
Stomach emptying	12.5	12.5	4.00	4.00
Duodenum	0.667	5.26	3.57	4.00
Jejunum	2.00	0.633	0.676	0.606
Ileum	4.167	25.0	16.7	0.787
Caecum	0.962	0.233	0.262	0.251
Colon	0.130	0.130	0.122	0.0838

Section S2: Deconvolution of the PK profiles

An apparent absorption rate constant k_a (i.e., model-independent) at each sampling time can be calculated based on the area-function method [58]:

$$k_a = \frac{C_{po}(t)}{F \cdot AUC_{iv}^{0-t} - AUC_{po}^{0-t}}$$

where $C_{po}(t)$ is the plasma concentration at time t after oral administration; F is the absolute bioavailability; AUC_{iv}^{0-t} and AUC_{po}^{0-t} is the AUC of intravenous and oral dose from time 0 to t.

The fraction of the amount absorbed at time t ($F_{a(t)}$) and the drug input rate ($dF_{a(t)}/dt$) can be expressed as:

$$F_a(t) = 1 - e^{-k_a t}$$

$$\frac{dF_a(t)}{dt} = k_a e^{-k_a t}$$

Following plots were obtained for the pharmacokinetics of IDP-73152 in mice, and rats (Figure S1).

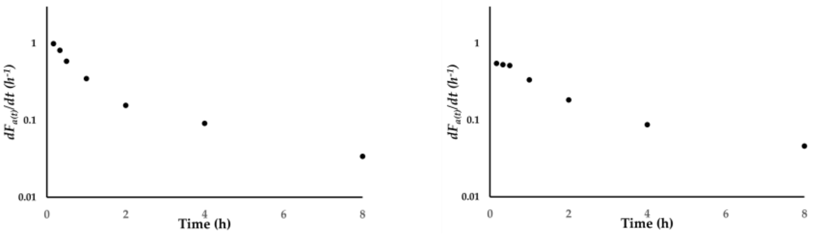

(**a**)	(**b**)

Figure S1. $dF_{a(t)}/dt$, i.e., the input rate, versus time plots for (a) mice, and (b) rats

Section S3: physicochemical properties

Table S3. Physiological properties of IDP-73152

Parameter	Value
Hydrogen bond donor count	3
Hydrogen bond acceptor count	5
Topological polar surface area	102 $Å^2$
log *P*	-0.52
p*K*a	8.8
